# Preferential Y-Y pairing and synapsis and abnormal meiotic recombination in a 47,XYY man with non obstructive azoospermia

**DOI:** 10.1186/s13039-016-0218-z

**Published:** 2016-02-02

**Authors:** Caiyun Wu, Liu Wang, Furhan Iqbal, Xiaohua Jiang, Ihtisham Bukhari, Tonghang Guo, Gengxin Yin, Howard J. Cooke, Zhenyi Cao, Hong Jiang, Qinghua Shi

**Affiliations:** 1 The Reproductive Medicine Center, Clinical College of People’s Liberation Army Affiliated to Anhui Medical University, Hefei, Anhui China; 2 The Reproductive Medicine Center, 105 Hospital of People’s Liberation Army, Hefei, Anhui China; 3 Molecular and Cell Genetics Laboratory, The CAS Key Laboratory of Innate Immunity and Chronic Diseases, Hefei National Laboratory for Physical Sciences at Microscale, School of Life Sciences, University of Science and Technology of China, Hefei, Anhui 230027 China; 4 Collaborative Innovation Center of Genetics and Development, Fudan University, Shanghai, 200438 China; 5 Institute of Pure and Applied Biology, Bahauddin Zakariya University, Multan, 60800 Pakistan; 6 Center for Reproductive Medicine, Anhui Medical University, Affiliated Provincial Hospital, Hefei, China; 7 Anhui Provincial Family Planning Institute of Science and Technology, Hefei, China

**Keywords:** Sex chromosomes, Meiosis, Meiotic sex chromosome inactivation (MSCI), XYY syndrome

## Abstract

**Back ground:**

Men with 47, XYY syndrome are presented with varying physical attributes and degrees of infertility. Little information has been documented regarding the meiotic progression in patients with extra Y chromosome along with the synapses and recombination between the two Y chromosomes.

**Methods:**

Spermatocyte spreading and immunostaining were applied to study the behavior of the extra Y chromosome during meiosis I in an azoospermia patient with 47, XYY syndrome and results were compared with five healthy controls with proven fertility.

**Results:**

The extra Y chromosome was present in all the studied spermatocytes of the patient and preferentially paired and synapsed with the other Y chromosome. Consistently, gamma-H2AX staining completely disappeared from the synapsed regions of Y chromosomes. More interestingly, besides recombination on short arms, recombination on the long arms of Y chromosomes was also observed. No pairing and synapsis defects between homologous autosomes were detected, while significantly reduced recombination frequencies on autosomes were observed in the patient. The meiotic prophase I progression was disturbed with significantly increased proportion of leptotene, zygotene cells and decreased pachytene spermatocytes in the patient when compared with the controls.

**Conclusions:**

These findings highlight the importance of studies on meiotic behaviors in patients with an abnormal chromosomal constitution and provide an important framework for future studies, which may elucidate the impairment caused by extra Y chromosome in mammalian meiosis and fertility.

**Electronic supplementary material:**

The online version of this article (doi:10.1186/s13039-016-0218-z) contains supplementary material, which is available to authorized users.

## Background

The 47,XYY sex chromosome variation is the most common sex chromosome anomaly after Klinefelter syndrome [1–3], occurring in approximately 1 out of 1000 live male births [4]. To account for the increased proportion of paternally derived 47,XXY males as compared to other trisomies it has been suggested that the XY bivalent, with its reduced region of homology, is particularly susceptible to non-disjunction [5]. Paternal non-disjunction at meiosis II resulting in sperm with an extra Y chromosome produces a 47,XYY karyotype in the affected offspring. Majority of the patients with 47,XYY have a delayed diagnosis, with a median age of 17.1 years at diagnosis [6]. Although most XYY boys have no phenotypic abnormalities, they are at greater risk for behavioral problems, mild learning disability, delayed speech/language development and usually with a tall stature [2].

An association between 47,XYY and fertility problems has been reported in several studies with an increased incidence of chromosomally abnormal spermatozoa in the semen of men with 47,XYY syndrome [7–13]. This greater prevalence of hyper haploid sperm results in an increased risk of passing the extra Y chromosome to offspring [2]. Men with 47,XYY syndrome can have variable sperm counts, ranging from normal to azoospermia [2, 10, 14–16].

Considerable attention has been given to the somatic abnormalities associated with 47,XYY conditions but less is known about their meiotic behaviors; that is, how sex chromosome imbalance influence the meiotic progression, and homologous pairing, synapsis and recombination. In this study, we applied Immnunofluorescence technique to study meiosis in a 47, XYY patient. We used immunostaining of SYCP3 and SYCP1 to study the sex chromosome configurations, MLH1 to detect recombination and γH2AX to determine meiotic sex chromosome inactivation (MSCI) in pachytene cells from the testis of our 47,XYY patient. We observed that the extra Y chromosome was present in all the studied spermatocytes of the patient, preferentially paired and synapsed with another Y chromosome, and associated with X chromosome, which may have affected the meiotic prophase I progression.

## Methods

### Patient and karyotype analysis

A 27 year old male was presented to Provincial Hospital affiliated with Anhui Medical University, Hefei, Anhui, People’s Republic of China. Semen analysis was carried out according to World Health Organization (WHO laboratory manual for the examination of human semen and semen-cervical interaction, 2010) and no sperm were observed in his semen. After obtaining written informed consent, testicular tissues were sampled from the patient. Five fertile men of Han ethnicity having at least one healthy child were recruited as normal controls for this study, and similar experiments were performed on them as mentioned for the patient. All the procedures of this study were approved by the institutional review board and ethical committee of the University of Science and Technology of China.

### Histological analysis

Testicular tissues were fixed overnight in 4 % PFA for histological examination. Serial testicular sections were made and positioned on microscope slides, stained with hematoxylin and eosin for histopathology analysis.

### Spermatocyte spreading and immunostaining

Testicular tissues were processed as we described previously [17, 18]. Rabbit anti-SYCP3 (Abcam, Cambridge, UK), human anti-CREST (Immunovision, Springdale, AR), mouse anti-MLH1 (BD Pharmingen Biosciences, San Diego, CA), mouse anti-γ-H2AX (Millipore, Billerica, MA) and Goat anti-SYCP1 (SantaCruz Biotechnology, CA, USA) were used as primary antibodies. These antibodies were detected using the following secondary antibodies: Alexa 555 donkey anti-rabbit (Molecular Probes, Carlsbad, CA), Alexa 488 goat anti-mouse (Molecular Probes, Carlsbad, CA), Alexa 488 donkey anti-goat (Molecular Probes, Carlsbad, CA), Alexa 488 donkey anti-mouse (Molecular Probes, Carlsbad, CA) and 1-amino-4-methylcoumarin-3-acetic acid (AMCA) donkey anti-human (Jackson Immunoresearch, West Grove, PA), respectively.

### Fluorescence in situ hybridization (FISH)

To identify the Y chromosomes in spermatocytes,,FISH was performed as we previously reported on the spermatocyte spreads immunostained for meiotic analyses in previous experiments using a DNA probe specific to the long arm of human Y chromosome(A generous gift from Professor Mingrong Wang, Cancer Institute and Hospital, Chinese Academy of Medical Sciences, Beijing, China). The Y probe was labeled with Spectrum Green dUTP (Vysis, 02N32-050) using a nick translation procedure following the manufacturer’s instructions. After the cover slips were moved, the slides were washed in PBS for 5 min, followed by dehydration in ethanol grades (70, 80, 90 and 100 %). After drying, the probes were added to the slides, co-denatured on a hotplate at 80 °C for 10 min and then the slides were incubated overnight in a humid chamber. Cover slips were moved and slides were washed in 0.4XSSC/0.3%NP-40 at 45°Cfor 45 min followed by 2XSSC/0.1 % NP-40 for 20 min at room temperature. After air drying in the dark, antifade and cover slips were added to the slides. Cells were analysed and imaged using an epifluorescence microscope Olympus BX61 (Olympus Inc., Tokyo, Japan) and Image Pro-Plus version 5.1 software (Media Cybernetics Inc., Bethesda, MD).

### Statistical analysis

Statistical analyses were performed using SPSS 13.0 software (SPSS Inc., Chicago, IL). A chi-square test was applied to compare the meiotic progression between the patient and controls. The Mann–Whitney test was applied for the comparison of MLH1 foci per cell between the patient and controls.

## Results

**Fig. 1:**
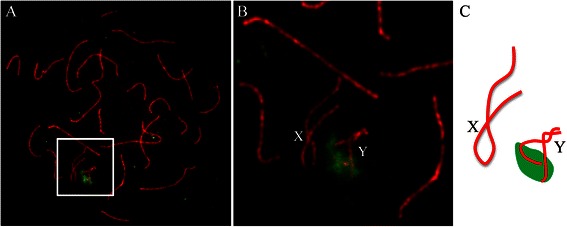
The extra Y chromosome was present in spermatocytes of the 47, XYY patient. **a** Image of a representative pachytene spermatocyte immunostained for SYCP3 (*Red*) showed two partially paired Y chromosomes identified by FISH using a DNA probe specific to the q arm of human Y chromosome (*Green*). **b** Enlarged area from A. **c** A schematic configuration of the sex chromosomes from the cell shown in **a** and **b**

Analysis of the semen revealed that the patient was suffering from azoospermia. Karyotyping on G-banded metaphases of peripheral blood lymphocytes revealed a karyotype of 47, XYY in all the 100 studied cells of the patient. FISH using a DNA probe specific to human Y chromosome on spermatocyte spreads indicated that all the 71 analyzed pachytene spermatocytes had a XYY constitution (Fig. 1).

### Prevalence of YY pairing and XY association in spermatocytes of the 47, XYY patient

**Fig. 2:**
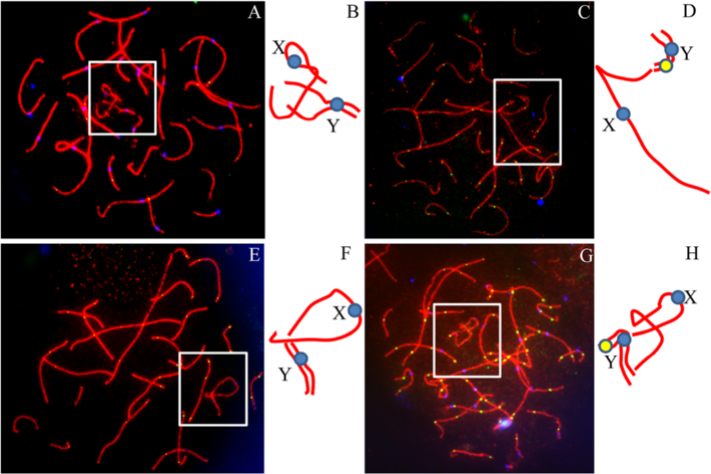
Sex chromosome pairing and recombination in the 47, XYY patient. Representative pachytene spermatocytes immunostained for CREST (*Blue*), SYCP3 (*Red*) and MLH1 (*Green*). **a** Two Y chromosomes partially paired and associated with X chromosome. There is no recombination on the sex chromosomes. **c** Two Y chromosomes partially paired and associated with chromosome X. Notably there is a recombination focus on the short arm of Y bivalent. **e** Two Y chromosomes completely paired and associated with X chromosome at the end of short arms. Notably there is no recombination on the sex chromosomes. **g** Two Y chromosomes completely paired and associated with chromosome X. Notably there is a recombination focus on the short arm of Y bivalent. **b**, **d**, **f** and **h** are the schematic configurations of the sex chromosomes from the cell shown in **a**, **c**, **e** and **g**, respectively

A total of 71 pachytene spermatocytes were analyzed for the pairing between homologous chromosomes. No abnormalities in pairing between homologous autosomes were observed in all the cells analyzed. For sex chromosomes, 42 out of 71 (or 59.2 %) cells showed pairing in whole Yp and partial Yq, while in the remaining 29 (or 40.8 %) cells the pairing extended to the whole length of Y chromosomes (Fig. 2; Table 1). Notably, in none of the studied pachytene cells, X was found to pair with Y (Fig. 2; Table 1).

**Table 1:** Sex chromosome pairing in pachytene spermatocytes in the 47, XYY patient

Cell type	No. of cells analyzed	No. (%^a^) of cells showing Y associated with X	No. (%^a^) of cells not showing Y associated with X
YY partially paired	42	27(64.3 %)^a^	15(35.7 %)^a^
YY completely paired	29	18(62.1 %)^a^	11(37.9 %)^a^
XY + Y	0	0	0
Total (%)	71	45(63.4 %)^a^	26(36.6 %)^a^

Sex chromosomes pairing was determined by over lapping SYCP3 signals

^a^The percentages were calculated by dividing the number of cells showing Y associated or not associated with X (respectively) with the corresponding number of cells analyzed

In more than 62.1 % spermatocytes analyzed, the Y chromosomes were found to be associated with X chromosome (Table 1). There was no significant difference in the frequency of cells showing XY association between partially and completely paired YY-containing cells (Table 1). In all the 45 spermatocytes displaying XY association, 36 (or 80 %), 3 (or 7 %) and 6 (or 13 %) cells showed an association of Xp with Yp, Xp with Yq and Xq with Yq, respectively.

### Abnormal sex chromosome synapsis the 47, XYY patient

**Fig. 3:**
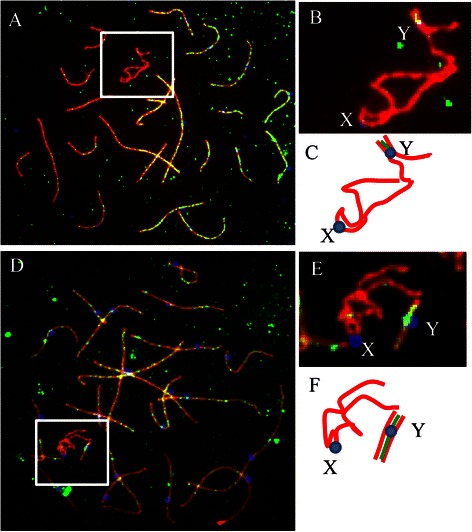
Synapsis between the two Y chromosomes in spermatocytes of the 47, XYY patient. Images of representative pachytene spermatocytes immunostained for CREST (*Blue*), SYCP3 (*Red*) and SYCP1 (*Green*). **a** Two Y chromosomes partially synapsed with one Y chromosome being associated with the X chromosome at the end of q arm. **b** Enlarged area from (**a**). **c** A schematic configuration of the sex chromosomes from the cell shown in **a** and **b. d**) Two Y chromosomes completely synapsed. **e** Enlarged area from (**d**). **f** A schematic configuration of the sex chromosomes from the cell shown in **d** and **e**

To detect synapsis between homologous chromosomes, SYCP1, a central element of synaptonemal complexes, were detected by immunostaining in pachytene spermatocyte spreads of the 47,XYY patient (Fig. 3). In all the 71 spermatocytes analyzed, no synapsis defects were observed for autosomes. For the two Y chromosomes, partialand complete synapsis was observed in 52 and 19 studied spermatocytes, respectively (Table 3). Higher frequency of Y-Y synapsis was observed in those spermatocytes where Y chromosomes were found associated with X chromosomes in all the 45 studied spermatocytes (Table 3). X chromosome was found associated with the Y chromosomes in 45 of the studied spermatocytes but synapses of X with Y chromosome was not observed in any of the studied pachytene spermatocytes (Fig. 3).

### Reduced recombination on sex chromosomes and autosomes of the 47, XYY patient

**Fig. 4:**
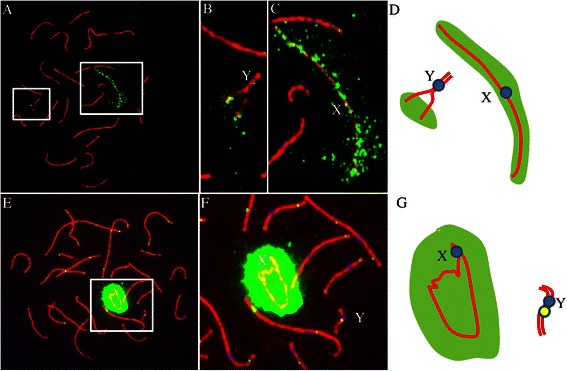
Unsynapsed regions of the sex chromosomes are stained positive for γH2AX while synapsed regions remained unstained in spermatocytes of 47,XYY male. Pachytene spermatocytes immunostained for γ-H2AX (*Green*), MLH1 (*Green*), CREST (*Blue*) and SYCP3 (*Red*). **a** γ-H2AX signals are not detected in the region of synapsed YY, but detected in unsynapsed regions of Y chromosomes and X chromosome. **b, c** Enlarged area from (**a**). **d** A schematic configuration of the sex chromosomes from the cell shown in **b** and **c. e** γ-H2AX signals are not visible on the completely synapsed YY but visible around X chromosome. **f** Enlarged area from (**e**). **g** A schematic configuration of the sex chromosomes from the cell shown in **e** and **f**

In order to determine effects of presence of two Y chromosomes on recombination during meiosis, the MLH1 foci, the meiotic recombination markers, were counted in pachytene spermatocytes of the patient. All the 65 spermatocytes with partial or complete YY synapsis were analyzed. Recombination between two the Y chromosomes was observed in 27 out of 65 or 41.5 % cells. In spermatocytes with partial YY synapsis, recombination was observed to occur between the two Yp in 21 out of 47 (or 44.7 %) cells (Fig. 2; Table 2), while in those with complete YY synapsis, recombination between the two Yp was seen as expected but at a lower frequency (4 out of 18, or 22.2 %, Table 2). More interestingly, recombination between the two Yq was also observed in 2 out of 18 (or 11.1 %) spermatocytes with complete YY synapsis (Fig. 4; Table 2). It was noted that the frequency of recombination between Yp arms was higher when two Y chromosomes were partially synapsed. More strikingly, MLH1 foci were not detected on remaining 38 cells although the two Y chromosomes were synapsed (Fig. 2; Table 2).

**Table 2:** Sex chromosome synapsis in pachytene spermatocytes in the 47, XYY patient

Cell type	No. of cells analyzed	No. (%^a^) of cells showing Y associated with X	No. (%^a^) of cells not showing Y associated with X
YY partially synapsed	52	35(67.3 %)^a^	17(32.7 %)^a^
YY completely synapsed	19	10(52.6 %)^a^	9(47.4 %)^a^
XY + Y	0	0	0
Total (%)	71	45(63.4 %)^a^	26(36.6 %)^a^

Sex chromosomes synapsis was determined by over lapping SYCP1 signals

^a^The percentages were calculated by dividing the number of cells showing Y associated or not associated with X (respectively) with the corresponding number of cells analyzed

Recombination frequency of autosomes in the 47, XYY patient was determined in71 pachytene spermatocytes and compared to those in 437 spermatocytes from five controls. The mean number of MLH1 foci per cell in the patient was significantly lower than those in the controls (44.9 ± 4.6, vs.48.1 ± 5.8; *P* <0.001, Mann–Whitney test).

### Meiotic sex chromosome inactivation (MSCI) in the 47, XYY, male

It has been documented that the chromosome or chromosome’s regions that had not experienced synapsis undergo inactivation and are decorated by γ-H2AX signals in spermatocytes [19, 20]. We immunostained mid-late pachytene spermatocytes of the patient and controls for the meiotic sex chromosome inactivation (MSCI) marker, phosphorylated H2AX (γH2AX), and the axial element protein SYCP3. In spermatocytes where two Y chromosomes were partially synapsed, γ-H2AX signals were detected around unsynapsed regions of two Y chromosomes and X chromosomes as expected (Fig. 4a-d). Interestingly, the γ-H2AX signals were only visible only on X chromosome in the spermatocytes where two Y chromosomes were completely paired and synapsed (Fig. 4e-g). These results indicate that synapsis can indeed occur between the two Y chromosomes.

### Meiotic progression was disturbed in 47, XYY male

**Fig. 5:**
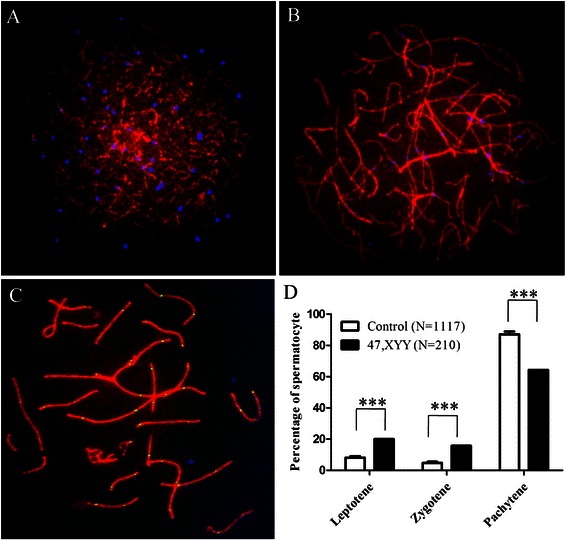
Meiotic progression was disturbed in the 47, XYY patient. Representative images show (**a**) Leptotene, (**b**) Zygotene and (**c**) Pachytene spermatocytes immunostained for SYCP3 (*Red*), MLH1 (*Green*) and CREST (*Blue*). **d** An increase in leptotene and zygotene, and decrease in pachytene spermatocytes were observed in the 47, XYY patient. N, The number of cells analysed; *** *P* <0.001, chi-square test

To determine whether the meiotic progression was distorted in our 47,XYY patient, a total of 210 spermatocytes in different sub-stages of meiotic prophase I were studied in the patient and the results were compared with the controls (1117 spermatocytes from 5 normal men). An increase in leptotene (*P* <0.001, chi-square test) and zygotene (*P* <0.001, chi-square test) but decrease in the pachytene spermatocytes (*P* <0.001, chi-square test) were observed in our patient (Fig. 5).

### Decreased germ cells in the testicular sections of the 47, XYY male

**Fig. 6:**
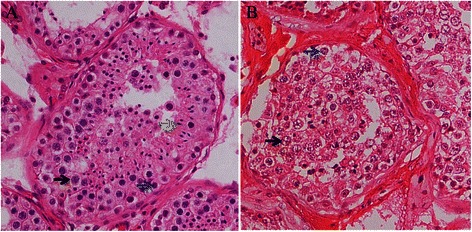
Decreased number of early germ cells and absence of sperm in the testicular sections of the 47, XYY male. H&E staining of testicular sections showed (**a**) normal histology and spermatogenesis in control and (**b**) reduced number of early germ cells and absence of sperm in the 47, XYY patient. *Blue arrow*, spermatogonia; *black arrow*, spermatocytes; *green arrow*, sperm

Histological examination of the H&E stained testicular sections revealed normal spermatogenesis with a lot of typical sperm in testicular tubules of a control male (Fig. 6a). However, reduced number of germ cells and no mature sperm were observed in testicular sections of the 47,XYY patient (Fig. 6b).

## Discussion

A lot of variation has been reported regarding the presence of the extra Y chromosome in germ cells, spermatogenesis and sperm counts in 47,XYY patients. Many men with 47,XYY karyotype has normal meiotic progression and are fertile. It has been suggested in some studies that the extra Y chromosome is lost before meiosis thus conserving fertility in these patients [2, 12, 15, 21]. In the present study, the extra Y chromosome was not lost in primary spermatocytes and XYY constitution was seen in all the 71 studied spermatocytes of our patient (Fig. 1; Table 1). This 47, XYY man showed disturbed meiotic progression and suffered from azoospermia with few germ cells in testicular tubules (Fig. 6b). Blanco et al. [22] reported that most (95.9 %) premeiotic cells and 57.9 % pachytenespermatocytes showed XYY chromosomal constitution, and 42.1 % of post-reductional germ cells were XY in their 47,XYY patient who was oligoasthenoteratozoospermia. Solari and Valzacchi [23] observed XYY constitution in all studied spermatocytes of a 47,XXY patient with severe oligozoospermia. Both of these studies concluded that the arrest point for the genetically abnormal germ cells may reside at the primary and secondary spermatocyte or spermatid stages leading to a continuous elimination of these cells during spermatogenesis. Solari and Valzacchi [23] observed a high level of germ cell death at or immediately after the meiotic divisions. Milazzo et al. [24] reported that two 47,XYY patients with severe oligozoospermia showed extra Y chromosome in 60.0 and 39.6 % of analyzed pachytene spermatocytes, respectively. They observed large number of apoptotic round spermatids and impaired meiotic division and cytokinesis failure leading to diploid (mainly 47,XYY cells) and tetraploid (94,XXYYYY) meiocytes, when present. Thus, the presence of the extra Y chromosomes in spermatocytes may disturb spermatogenesis and result in infertility of 47,XYY males.

We have observed abnormal pair and synapsis of sex chromosomes in our 47,XYY patient. A predominant pair and synapsis pattern observed was a partially or completely paired and synapsed YY bivalent associated with X chromosome, which forming a trivalent in 45 of 71, or 63.4 % of studied spermatocytes (Figs. 2 and 3; Tables 1 and 3). The minor pair and synapsis pattern was an YY bivalent and a univalent X in 26 of 71, or 36.6 % of the studied cells, while in none of the studied cells, X and Y were found associated with each other (Figs. 2 and 3; Tables 1 and 3). Several other studies on pachytene cells have also reported that the two Y-chromosomes preferentially pair and synapsis [2, 22, 23]. Solari and Valzacchi [23] had found a complete absence of normal XY pachytene spermatocytes and 86 % spermatocytes showed Y-Y bivalent plus a univalent X in their XYY patient. We thus conclude that pair and synapsis occur preferentially between two Y chromosomes to form X+YY configuration in XYY spermatocytes.

**Table 3:** Recombination on YY bivalents in the 47, XYY patient

Cell type	No. of cells analyzed	No. (%^a^) of cells with recombination in Yp	No. (%^a^) of cells with recombination in Yq	Total recombination in YY bivalent (%)
YY partially synapsed	47	21 (44.7 %)^a^	0	44.7%^a^
YY completely synapsed	18	4(22.2 %)^a^	2 (11.1 %)^a^	33.3%^a^
Total (%)	65	25 (38.5 %)^a^	2 (3.0 %)^a^	41.5%^a^

Sex chromosomes recombination was determined by MLH1 signals

^a^The percentages were calculated by dividing the number of cells showing recombination on Y chromosomes with the number of cells analyzed

The preferential pairing and synapsis of the Y-chromosomes is may be due to their greater homology compared with the X chromosome. X+YY cells are likely to be lethal due to the escape of Y genes and prevention of X genes from meiotic sex chromosome inactivation (MSCI), which normally silences the unsynapsed sex chromosomes [25] and result in a low sperm count [2, 23, 24, 26]. In consistence, we observed that synapsis took place along at least entire short arms and partial long arms of the two Y chromosomes, but never between X and Y chromosomes, in all the studied pachytene spermatocytes (Fig. 3), and the γ-H2AX signals were only visible on X and distal part of Yq (Fig. 4). γ-H2AX signals were not detected in the region where YY were synapsed YY while γ-H2AX signals were detectable in unsynapsed regions of Y and X chromosomes (Additional file 1: Figure S1) indicating that synapsis of the Y chromosomes can afford protection of the Y chromosome from γ-H2AX phosphorylation preventing MSCI.

It has been established that each pair of homologous chromosomes must have at least one recombination between them, and the recombination only occurs on the pseudo autosomal region (PAR) of X and Y chromosomes in normal spermatocytes [27, 28]. In our 47,XYY patient, we observed recombination between two Y chromosomes in 27 of 65, or 41.5 % cells analyzed, in which 25 spermatocytes showed recombination between two Yp arms while 2 showed recombination between two Yq arms. This indicates at least that long arm of Y chromosome also has the property to pair, synapse and recombine between each other during meiosis. MLH1 foci were not detected on remaining 38 cells although the two Y chromosomes were paired (Fig. 2; Table 2), the reason for this remains unknown. To our surprise, the number of MLHI foci on autosomes were also significantly reduced in our 47,XYY patient when compared to the controls (Table 2), which indicates presence of an inter-chromosomal effect (ICE). This ICE was probably due to activation of pachytene checkpoint that delays pachytene progression when either the process required for crossover is defective or when there are defects in the structure of meiotic chromosome axis [29].

A huge variation in number of MLH1 foci has been reported in subjects suffering from male infertility (with variety of phenotypes) when compared with the normal fertile controls. It has been reported previously that status and age of the subjects did not affect the recombination frequencies. Lynn et al. [30] did not find any difference in recombination frequencies based on patient status (e.g., cancers, cystic fibrosis, or previous vasectomy) and age. This study shows that the control data we have used in our manuscript is reliable and can be used to compare the recombination frequencies between the patient and controls. Regarding the counts of MLH1 foci in control and patient spermatocytes and the % of cells with MLH1 on XY chromosomes, individual variations has been reported for both patients and controls. Sun et al. [31] has reported mean frequency of 49.8 ± 4.3 of autosomal recombination foci in a 47 year old control having 73 % recombination focus in XY bivalent. While Codina-Pascual et al. [32] has studied the meiotic progression in 4 patients with azoospermia and 6 patients suffering from oligoasthenozoospermia. They have reported that the mean MLH1 foci per cell were similar in patients (47.3) and controls (48.8). Upon comparison of MLH1 foci in sex chromosomes they observed decreased mean MLH1 foci (% cells) in patients (59.2 %) as compared to controls (69.9 %). It was observed that number of MLH1 foci on X-Y chromosome was different in patients suffering from azoospermia (61.7 %) and oligoasthenozoospermia (59.2 %) indicating that MLH1 foci number varies with the underlying type of infertility. Sun et al. [33] has also reported decreased mean MLH1 foci (% cells) in 7 patients with non obstructive azoospermia (77.7 %) as compared to controls (86.2 %). The mean number of MLH1 foci (% cells) in this study is higher for both patients and controls than the data we have presented in the submitted manuscript and Sun et al. [33] has reported that despite having 77.7 % MLH1 foci on sex chromosomes, the patients suffered from reduced meiotic recombination on the XY bivalent confirming that there is no specific range for MLH1 foci per cell for both autosomes as well as sex chromosomes and the number of MLH1 foci varies from person to person.

## Conclusion

In conclusion, we have reported that the extra Y chromosome was present in all the studied pachytene spermatocytes of a 47, XYY patient. Presence of this extra Y chromosome has resulted in abnormal sex chromosome pairing, synapsis and recombination and prevented the meiotic sex chromosome inactivation, leading to disturbed spermatogenesis, germ cell loss and consequently azoospermiain our patient. Hence, this work provides an important framework for future studies, which may elucidate the impairment caused by extra Y chromosome in mammalian meiosis and fertility.

## Additional file

Additional file 1: Figure S1. Unsynapsed regions of the sex chromosomes are stained positive for γH2AX while synapsed regions remained unstained when the YY associated with X chromosome in spermatocytes of 47,XYY male. Pachytene spermatocytes immunostained for γ-H2AX (Green), MLH1 (Green), CREST (Blue) and SYCP3 (Red). (A) γ-H2AX signals were not detected in the region where YY were synapsed YY while γ-H2AX signals were detectable in unsynapsed regions of Y and X chromosomes. (B) Enlarged area from (A). (C) A schematic configuration of the sex chromosomes from the cell shown in B. white arrow indicating the region with synapsed YY. (TIF 4775 kb)
