# Supplementary figures and images for: Preferential Y-Y pairing and synapsis and abnormal meiotic recombination in a 47,XYY man with non obstructive azoospermia
Source: Mol Cytogenet. 2016 Feb 2;9:9. doi: 10.1186/s13039-016-0218-z (PMC4736128; doi:10.1186/s13039-016-0218-z)

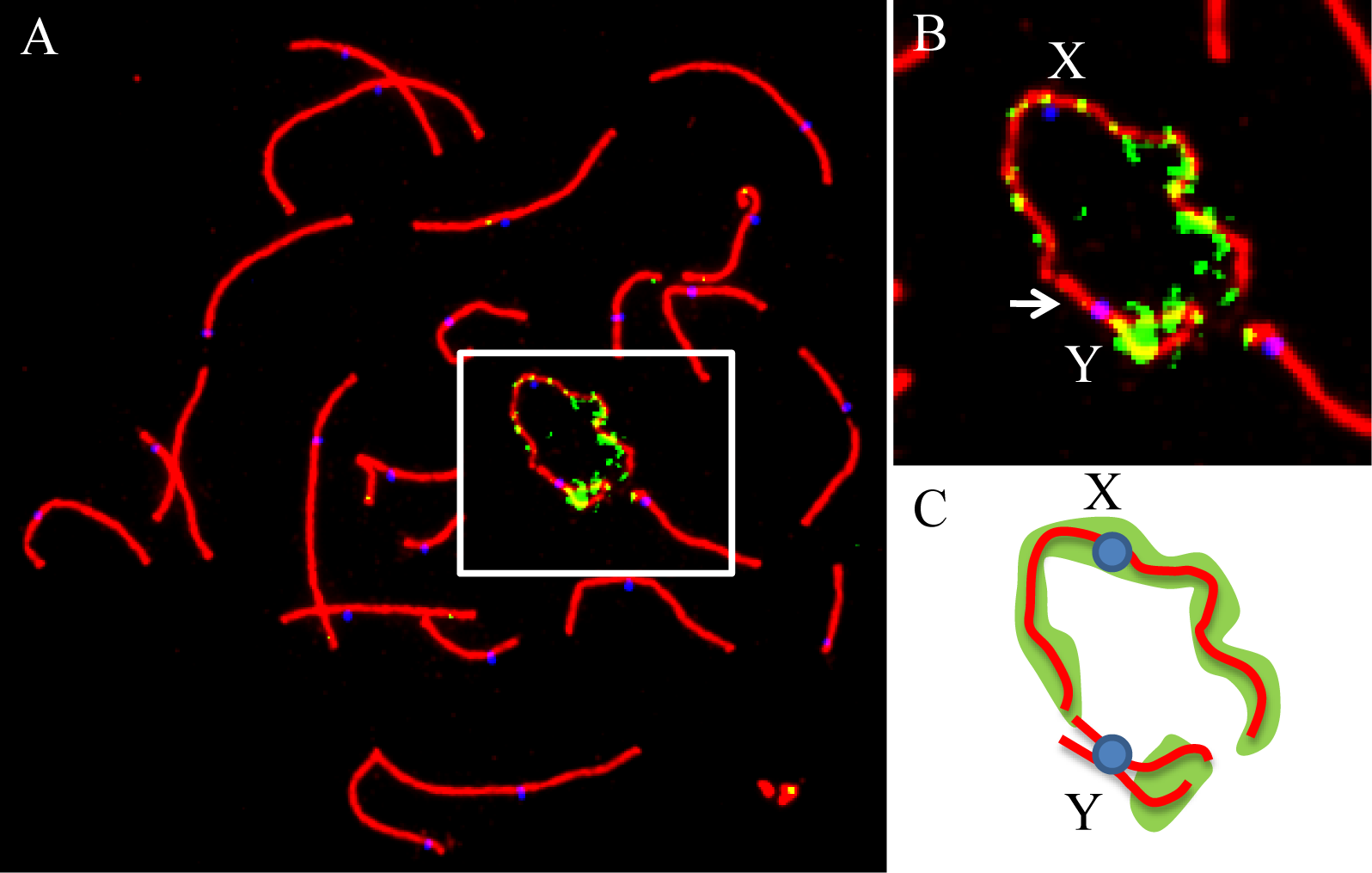

Supplement: Additional file 1: Figure S1. — Unsynapsed regions of the sex chromosomes are stained positive for γH2AX while synapsed regions remained unstained when the YY associated with X chromosome in spermatocytes of 47,XYY male. Pachytene spermatocytes immunostained for γ-H2AX (Green), MLH1 (Green), CREST (Blue) and SYCP3 (Red). (A) γ-H2AX signals were not detected in the region where YY were synapsed YY while γ-H2AX signals were detectable in unsynapsed regions of Y and X chromosomes. (B) Enlarged area from (A). (C) A schematic configuration of the sex chromosomes from the cell shown in B. white arrow indicating the region with synapsed YY. (TIF 4775 kb) [file 13039_2016_218_MOESM1_ESM.tif]
